# Supplementary material for: Adipocyte ALK7 links nutrient overload to catecholamine resistance in obesity
Source: eLife. 2014 Aug 26;3:e03245. doi: 10.7554/eLife.03245 (PMC4139062; doi:10.7554/eLife.03245)
Supplement: Supplementary file 1. — Primers for qPCR. Sequences of primers used for qPCR are given. DOI: http://dx.doi.org/10.7554/eLife.03245.019 [file elife03245s001.docx]

**Supplementary file 1. Primers for qPCR**

| **Gene** |  | **Sequence** |
| --- | --- | --- |
| *Adrb1* | Forward Primer | CTCATCGTGGTGGGTAACGTG |
|  | Reverse Primer | ACACACAGCACATCTACCGAA |
| *Adrb2* | Forward Primer | GGGAACGACAGCGACTTCTT |
|  | Reverse Primer | GCCAGGACGATAACCGACAT |
| *Adrb3* | Forward Primer | AGAAACGGCTCTCTGGCTTTG |
|  | Reverse Primer | TGGTTATGGTCTGTAGTCTCGG |
| *Alk7* | Forward Primer | GCT TTC CAT AGC GAG TGG TC |
|  | Reverse Primer | ATC TCG GTG AGC AAT AGC AG |
| *Cytochrome C* | Forward Primer | CCAAATCTCCACGGTCTGTTC |
|  | Reverse Primer | ATCAGGGTATCCTCTCCCCAG |
| *Elovl3* | Forward Primer | GCCTCTCATCCTCTGGTCCT |
|  | Reverse Primer | TGCCATAAACTTCCACATCCT |
| *Hadhb* | Forward Primer | ACTACATCAAAATGGGCTCTCAG |
|  | Reverse Primer | AGCAGAAATGGAATGCGGACC |
| *HSL* | Forward Primer | CCAGCCTGAGGGCTTACTG |
|  | Reverse Primer | CTCCATTGACTGTGACATCTCG |
| *Gdf3* | Forward Primer | CCGAGTTTCAAGACTCTGACC |
|  | Reverse Primer | CGAGCCCGGATGATTTTCCTT |
| *PGC1alpha* | Forward Primer | TATGGAGTGACATAGAGTGTGCT |
|  | Reverse Primer | CCACTTCAATCCACCCAGAAAG |
| *PPARg* | Forward Primer | TCGCTGATGCACTGCCTATG |
|  | Reverse Primer | GAGAGGTCCACAGAGCTGATT |
| *Rgs2* | Forward Primer | GAGAAAATGAAGCGGACACTCT |
|  | Reverse Primer | GCAGCCAGCCCATATTTACTG |
| *UCP1* | Forward Primer | GGCCTCTACGACTCAGTCCA |
|  | Reverse Primer | TAAGCCGGCTGAGATCTTGT |
| *UCP3* | Forward Primer | CTGCACCGCCAGATGAGTTT |
|  | Reverse Primer | ATCATGGCTTGAAATCGGACC |
